# ORCA: A picture database of object–scene arrangements for cross-cultural and aging research

**DOI:** 10.3758/s13428-023-02064-x

**Published:** 2023-01-26

**Authors:** Michael Weigl, Jan Pietsch, Efsevia Kapsali, Qi Shao, Zhiwei Zheng, Juan Li, Jutta Kray, Axel Mecklinger

**Affiliations:** 1 Department of Psychology, Saarland University, Campus A2.4, D-66123 Saarbrücken, Germany; 2 CAS Key Laboratory of Mental Health, Institute of Psychology, Chinese Academy of Sciences, Beijing, 100101 China; 3 Department of Psychology, University of Chinese Academy of Sciences, Beijing, 100049 China

**Keywords:** Culture, Aging, Familiarity, Semantic congruency, Image database

## Abstract

In recent years, cross-cultural research on the modulation of basic cognitive processes by culture has intensified – also from an aging perspective. Despite this increased research interest, only a few cross-culturally normed non-verbal stimulus sets are available to support cross-cultural cognitive research in younger and older adults. Here we present the ORCA (Official Rating of Complex Arrangements) picture database, which includes a total of 720 object–scene compositions sorted into 180 quadruples (e.g., two different helmets placed in two different deserts). Each quadruple contains visually and semantically matched pairs of objects and pairs of scenes with varying degrees of semantic fit between objects and scenes. A total of 95 younger and older German and Chinese adults rated every object–scene pair on object familiarity and semantic fit between object and scene. While the ratings were significantly correlated between cultures and age groups, small but significant culture and age differences emerged. Object familiarity was higher for older adults than younger adults and for German participants than for Chinese participants. Semantic fit was rated lower by German older adults and Chinese younger adults as compared to German younger adults and Chinese older adults. Due to the large number of stimuli, our database is particularly well suited for cognitive and neuroscientific research on cross-cultural and age-related differences in perception, attention, and memory.

**Supplementary Information:**

The online version contains supplementary material available at 10.3758/s13428-023-02064-x.

## Introduction

Several decades of cross-cultural research have revealed consistent differences in perceptual and cognitive processing between East Asian and Western cultures. For example, Westerners (e.g., American, British, or German people) who live in an individualistic society exhibit an independent self-construal and an analytic thinking style, while East Asians (e.g., Chinese or Japanese people) who live in collectivistic societies have an interdependent self-construal and a holistic thinking style (Choi et al., 2007; Nisbett, 2003; Singelis & Sharkey, 1995; Varnum et al., 2010).

Cultural differences, however, are not restricted to the level of attitudes or inter-personal behavior. Mounting evidence suggests that cultural differences extend even to the basic perceptual and cognitive processes such as causal attribution, categorization, scene perception, attention allocation, and memory (Nisbett, 2003; Nisbett & Masuda, 2003). Regarding attentional processing, it is well documented that Westerners show a stronger focus on objects and less focus on the context than East Asians (Masuda, 2017) as evidenced, for example, by eye-tracking studies (e.g., Chua et al., 2005; Masuda et al., 2008).

Many cross-cultural memory studies have investigated differences in analytic vs. holistic processing by showing pictures of objects or persons in front of a background scene (Chua et al., 2005; Evans et al., 2009; Ko et al., 2011; Masuda et al., 2008; Masuda & Nisbett, 2001; Mickley Steinmetz et al., 2018). In later recognition tests, memory is probed for either central stimulus aspects, peripheral stimulus aspects, or both. Typically, recognition memory for the central object is more hampered by peripheral information in East Asians than in Westerners (e.g., Chua et al., 2005; Masuda et al., 2008; Masuda & Nisbett, 2001; Mickley Steinmetz et al., 2018). At the same time, East Asians showed superior memory for background information relative to Westerners (e.g., Ko et al., 2011). These results support the view that East Asians adapt a more holistic and Westerners a more analytic processing style, even though these patterns are not found in every study (e.g., Evans et al., 2009).

One major problem with some of the aforementioned studies (e.g., Chua et al., 2005; Evans et al., 2009; Masuda & Nisbett, 2001) is that the applied stimulus sets rarely exceed a few dozen images and often no ratings for critical stimulus features (such as lifetime familiarity; Souza et al., 2020) are reported for the cultures under comparison. In other cases, ratings are available for parts of the stimulus (e.g., valence and arousal for International Affective Picture System background images; Lang & Bradley, 2005), but not for the stimulus combination used in the study (e.g., Ko et al., 2011). For cross-cultural research, stimulus material rated by members of two or more cultures are necessary in order to obtain valid and informative results about cultural differences and human universalities (e.g., Yoon et al., 2004).

Age is another factor, which tremendously affects cognitive performance. Ample research has demonstrated that cognitive mechanics (i.e., basic processes such as processing speed or working memory) decline with age (see Park & Gutchess, 2006, for a short review). Domains related to the cognitive pragmatics (i.e., acquired knowledge), in contrast, show a modest increase with age (e.g., Park & Gutchess, 2006). For example, episodic memory is particularly affected by age even in healthy adults, with memory for associations (e.g., an object in front of a background scene) being more affected than memory for items itself (e.g., the particular object) – a phenomenon known as the associative memory deficit (Naveh-Benjamin, 2000; Naveh-Benjamin & Mayr, 2018). Associative memory deficits have been observed for verbal and non-verbal materials (see Old & Naveh-Benjamin, 2008 for a meta-analysis). Moreover, and relevant for the present study, when the separate items can be encoded as a single unit (i.e. unitization), older adults can learn pictorial associations without semantic relations despite their associative deficit (Bridger et al., 2017; Huffer et al., 2022). However, age effects are not restricted to memory. For instance, older adults have more difficulties in executing cognitive control during visual search than younger adults (Borges et al., 2020). For aging research, too, pictorial stimulus material rated by younger and older adults is helpful to allow for valid conclusions about the impact of age, which are unbiased by age-related changes in the perception of the stimulus material. Yet, the majority of pictorial stimulus sets provide ratings only for younger adults (see Souza et al., 2020, for a review).

Group-specific ratings are especially relevant for research examining age by culture interactions, as there is evidence that age-related changes modulate the magnitude of cultural differences in cognition (Park & Gutchess, 2006). More specifically, Park et al. (1999) suggested that cultural differences in cognitive pragmatics increase with age as more culture-specific experiences are accumulated over the course of a lifetime. By contrast, cultural differences in cognitive mechanics decrease with age, because the age-related decline of cognitive functions leads to an assimilation of performance across cultures (Park et al., 1999; Park & Gutchess, 2006). The differential effect of culture and age on cognitive pragmatics vs. mechanics is one example that highlights the need for stimulus material with culture-specific norms not only for younger, but also for older adults.

Here we present the ORCA (Official Rating of Complex Arrangements) picture database, which we created with the following five criteria in mind taking into account the special requirements on cross-cultural aging research.

First, there should be object–scene compositions similar to Masuda and Nisbett (2001) or Chua et al. (2005) with a visually and semantically matched distractor for every object and scene. This means that four object–scene compositions formed a quadruple with two visually and semantically matched object and two visually and semantically matched scenes (e.g., two types of red helmets placed in two different desert scenes). A high similarity between the two stimuli not only facilitates counterbalancing, but also the implementation of experimental manipulations to investigate memory. The high feature overlap is particularly useful for tapping into hippocampus-based mnemonic processes (Stark et al., 2019). Moreover, a high similarity might also prevent the use of information reduction strategies (e.g., sole reliance on the color of the stimulus), which could bias memory retrieval.

Second, there should be a varying degree of semantic fit between the objects and scenes (i.e., an association between object and scene or an expectation of encountering the object in the scene) to allow for the study of effects of congruency and incongruency. Since we wanted to investigate cultural differences in the mnemonic binding of arbitrary object–scene combinations, we aimed for a stimulus set with a high number of object–scene compositions with low semantic fit. In addition, a low fit between object and scene would prevent preexisting knowledge to guide associative memory decisions.

Third, the material should be rated by younger and older German and Chinese adults. Germans and Chinese have long-standing differences in their intellectual traditions (Nisbett, 2003). Germans as a Western culture analytic intellectual tradition trace their analytic intellectual tradition back to ancient Greece, where philosophers detached objects from its context and relied on formal logic as tools for analysis. The Chinese intellectual tradition is based on holistic philosophies from Taoism, Confucianism, and Buddhism, which place a strong emphasis on the context and strive for a middle way between opposing views. Thus, Germans and Chinese can be viewed as representatives of Western and East Asian cultures, respectively (Hofstede, 2001; Nisbett, 2003).

Fourth, the included objects should be familiar to both cultures and age groups. Lifetime familiarity is known to affect memory (Mecklinger & Bader, 2020) and substantial differences in familiarity with the objects between the groups could systematically bias object recognition and memory for the object. Moreover, it has been shown that cultural differences in perceptual experience can already affect early stages of visual processing (Mecklinger et al., 2014). Thus, we collected familiarity ratings to ascertain that both cultures and age groups are highly familiar with the depicted object.

Fifth, there should be a large number (i.e., several hundreds) of stimuli. This is especially relevant for neuroscientific research (e.g., EEG or fMRI experiments), which often requires a large number of trials to achieve a satisfactory signal-to-noise ratio (Luck, 2014). In addition, eye-movement artifacts can greatly distort neurophysiological data. Trials containing eye-movement artifacts often need to be discarded or corrected with computation-intensive methods (Jung et al., 2000). In order to reduce eye-movement artifacts during recording in neuroscientific studies, all objects were placed in the middle of the scene and easy to spot. To the best of our knowledge, no picture set currently available in the literature satisfies all of the above-mentioned criteria.

To date, there already exist some valuable sets of cross-culturally rated pictorial stimuli in the literature (see Souza et al., 2020, for a review). For example, several norms have been published for the widely used Snodgrass and Vanderwart (1980) set of pictures, which are available as black and white line drawings (Snodgrass & Vanderwart, 1980) and as gray-level and colored line drawings (Rossion & Pourtois, 2004). For example, E. Bates et al. (2003) provided timed picture naming norms for seven languages (English, Spanish, Italian, German, Hungarian, Bulgarian, and Chinese). Yoon et al. (2004) normed the Snodgrass and Vanderwart (1980) picture set not only for American and Chinese younger adults, but also for American and Chinese older adults. Recently, the MultiPic set of 750 colored line drawings, which was rated in English, Spanish, Italian, French, German, and Dutch, has been added to the pool of available picture sets (Duñabeitia et al., 2018). However, the stimuli of these data sets rely on drawing whereas it has been documented in the meanwhile that memory is better for realistic scenes than line drawings (Brodeur et al., 2017; Loftus & Bell, 1975).

More recently, there are stimulus sets with realistic scenes that contain congruent and incongruent object–scene compositions. One such stimulus set is the SCEGRAM database (Öhlschläger & Võ, 2017). SCEGRAM contains 62 scenes with semantically consistent or inconsistent objects placed in physically possible and impossible locations. However, the material was only rated by German participants so that some stimuli may be rather specific to the German culture. Also, the number of different scenes is rather low, which might be problematic for application in neuroscientific research. Another picture set, the Berlin Object in Scene (BOiS) database, contains 130 scenes with or without semantically related object at expected and unexpected locations (Mohr et al., 2016). A key feature of BOiS is that photos were shot with the objects actually placed in the scene. This resulted in very naturalistic scenes. However, it is often difficult to spot and discern the target object from the background as BOiS was created for studies on visual search. This renders BOiS less suitable for memory studies.

In sum, culturally normed data sets of picture sets mostly rely on line drawings that are less suitable for memory research than realistic scenes, while data sets with realistic scenes are not normed for different cultures and age groups. Hence, there are no ratings for German and Chinese participants available for objects on different scenes, which would be particularly useful for cross-cultural studies on memory. Moreover, most data sets consist of a rather small number of stimuli which is a strong limitation for any cognitive research. With ORCA, we provide a cross-culturally normed picture set which addresses these issues and is especially suited for cross-cultural aging research from a neuroscience perspective.

## Method

### Participants

In total, 24 younger German, 23 older German, 24 younger Chinese, and 24 older Chinese adults participated in our study (see Table 1 for more information on the sample characteristics)^1^. Younger German and Chinese students were recruited at the Saarland University in Saarbrücken (Germany) and the University of the Chinese Academy of Sciences in Beijing (China), respectively. Older German and Chinese participants were recruited from the Saarland and Beijing residential area, respectively, from participant databases or via advertisement in newspapers or social media. Interested participants were only tested if they had no known neurological or psychiatric disease, normal or corrected-to-normal vision, identified with the culture at the test location, and were 18–30 (younger adults)/65–80 (older adults) years old. Participants received money or partial course credit as compensation for participation. The experimental procedures were approved by the Ethics Committee of the Institute of Psychology, Chinese Academy of Sciences and the Ethics Committee of the Faculty of Human and Business Sciences at Saarland University.

**Table 1:** Demographic information on our sample

	German		Chinese	
	Younger adults	Older adults	Younger adults	Older adults
*N*	24	23	24	24
Mean age (*SD*)	22.7 (2.6)	71.8 (3.8)	21.8 (2.4)	70.1 (4.1)
Age range	19–28	65–80	19–29	65–79
Gender ratio	13 F/11 M	14 F/10 M	12 F/12 M	16 F/8 M
Years of education	13.9 (3.6)	13.4 (4.0)	15.7 (1.6)	11.6 (2.3)
SCS Independence	4.20 (0.42)	4.52 (0.51)	3.83 (0.38)	4.43 (0.84)
SCS Interdependence	3.87 (0.45)	3.79 (0.52)	3.96 (0.49)	4.60 (0.90)
Mean MMSE (*SD*)	-	28.83 (1.15)	-	28.88 (1.19)

*SD* standard deviation; *SCS* self-construal scale; *MMSE* Mini-Mental State Examination

Our German and Chinese samples were comparable in terms of age within their age group (younger adults: *t*(46) = –1.20, *p* = .235, Cohen’s *d* = .35, older adults: *t*(45) = –1.51, *p* = .138 Cohen’s *d* = .44) and gender (χ^2^(4) = 1.60, *p* = .808). The scores of older adults on the Mini-Mental State Examination (MMSE; a short test for screening for dementia-related cognitive impairment; Folstein et al., 1975) were also comparable (*t*(45) = –0.14, *p* = .887, Cohen’s *d* = .04) and all participants scored 26 points or higher (out of 30 points; scores of 26 or above indicate normal cognitive functioning; Zheng et al., 2015). Please note that age was not considered for scoring.

The analysis of variance (ANOVA) for years of education revealed a main effect for Age Group (*F*(1, 90) = 13.42, *p* < .001, η_p_^2^ = .13), indicating that the younger participants had more years of education than the older participants. There was no main effect for Culture (*F*(1, 90) = 0.00, *p* = .957, η_p_^2^ = .00). Moreover, there was an interaction between Culture and Age Group (*F*(1, 90) = 8.45, *p* = .005, η_p_^2^ = .09). Follow-up *t* tests revealed an age effect in years of education for the Chinese sample (*t*(46) = 7.18, *p* < .001, Cohen’s *d* = 2.07), indicating that the older Chinese participants spent less time in education than the younger Chinese participants. This was not the case for the German participants (*t*(44) = 0.42, *p* = .678, Cohen’s *d* = .12). Please note that the term years of education (“Jahre der schulischen/akademischen Ausbildung”) may have been misleading for some German participants. For this reason, some participants wrote down only the number of years in higher education despite 12–13 years at public school. Therefore, the results for the German sample should be treated with caution.

The Self-Construal Scale (SCS; Kitayama et al., 2014; Singelis, 1994; Singelis & Sharkey, 1995), a widely used scale to measure independent and interdependent self-construal, was used to test for cultural differences. Typically, East Asians score lower on independent SCS and/or higher on interdependent SCS than Westerners (Singelis & Sharkey, 1995; Yoon et al., 2000). The SCS served as a “manipulation check”, i.e., we wanted to ascertain that the participants in our sample are representative for their culture. For the independent SCS, we found no significant effect for Culture (*F*(1, 91) = 3.78, *p* = .055, η_p_^2^ = .04), but a main effect for Age Group (*F*(1, 91) = 15.59, *p* < .001, η_p_^2^ = .15), indicating that self-construal was more independent for older adults than for younger adults. The interaction between Culture and Age Group was not significant (*F*(1, 91) = 1.46, *p* = .231, η_p_^2^ = .02). Given the potential of a type II error, we had a closer look at the results. While the ANOVA would suggest that culture did not play a role for the independent SCS, group-specific analyses reveal a different pattern. In fact, German younger adults had a more independent self-construal than Chinese younger adults (*t*(46) = 3.18, *p* = .003, Cohen’s *d* = .92). This was not the case for older adults (*t*(45) = 0.42, *p* = .676, Cohen’s *d* = .12).

For the interdependent SCS, we found a significant main effect for Culture (*F*(1, 91) = 12.67, *p* < .001, η_p_^2^ = .12), a significant main effect for Age Group (*F*(1, 91) = 5.03, *p* = .027, η_p_^2^ = .05), and a significant interaction between Culture and Age Group (*F*(1, 91) = 7.92, *p* = .006, η_p_^2^ = .08). There was a significant cultural difference in interdependent self-construal for older adults (*t*(45) = –3.75, *p* < .001, Cohen’s *d* = 1.09), indicating that Chinese older adults had a more interdependent self-construal than German older adults. This was not the case for younger adults (*t*(46) = –0.70, *p* = .490, Cohen’s *d* = .20).

To sum up, our German and Chinese samples are comparable in terms of age, gender, and cognitive functioning. Years of education were similar for younger and older German participants, whereas older Chinese participants had fewer years of education than younger Chinese participants. Furthermore, the results from the SCS provided evidence for the expected cultural differences in our sample. This led us to conclude that our sample is representative and comparable enough to ensure the validity of the picture ratings.

### Materials

We created 180 object pairs and 180 scene pairs based on physical and conceptual similarity. Objects were taken from Brady et al. (2008) (https://bradylab.ucsd.edu/stimuli.html) and scenes from a database from Goh (2010)^2^. Additional objects and scenes were found via Google Image Search and Pixabay (https://pixabay.com). All objects were PNG images of the same size (256 x 256 pixels). The size of all scenes was the same (640 x 480 pixels). Physical and conceptual similarity between the two objects and between the two scenes were determined by the authors and qualitatively evaluated by an informal committee consisting of the authors and student assistants from both labs (six persons on the German side and five persons on the Chinese side). In case of disagreements, the stimulus material was replaced until both sides agreed on the selection. Moreover, care was taken that the objects were familiar and that scenes were meaningful to both cultures. Again, objects and scenes were replaced until all members of the committee agreed on the selection and the pairing.

**Fig. 1:**
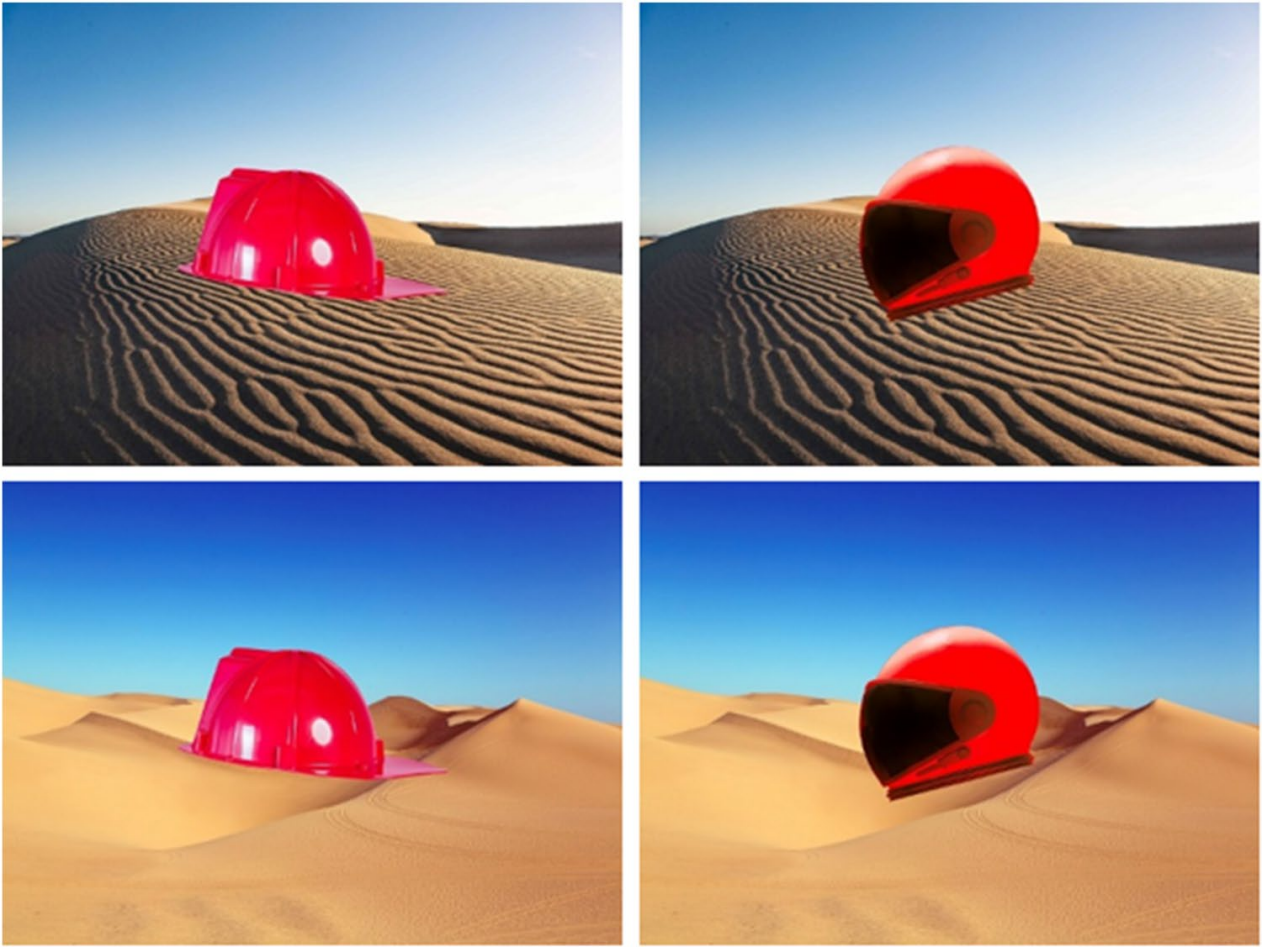
Examples for the composition of our stimulus set. We created quadruples with two objects, which should be familiar to the participants, shown in front of two different scenes. This is an example of a quadruple without semantic fit. We placed two different helmets in two different desserts

Next, we placed 150 object pairs on semantically unrelated scene pairs and 30 object pairs on semantically related scene pairs using Photoshop CS6 and GIMP 2.10. For the purpose of the study, we defined semantic fit as an association between object and scene or as expectation of encountering the object in the scene. Each object was placed in the center of the scene. Again, (un-)relatedness was determined by the authors and qualitatively evaluated by the informal committee. Objects were placed on different backgrounds until all members of the committee agreed to the selection. Thus, we had 180 quadruples and a total of 720 scene–object arrangement (640 x 480 pixels). An example for a quadruple can be seen in Fig. 1. The material can be downloaded here: https://www.uni-saarland.de/fileadmin/upload/lehrstuhl/mecklinger/Dokumente/ORCA.zip.^3^ Information on the physical properties of the stimuli can be found in the accompanying Excel file (see S1 for more information on the Excel table).

### Procedure

**Fig. 2:**
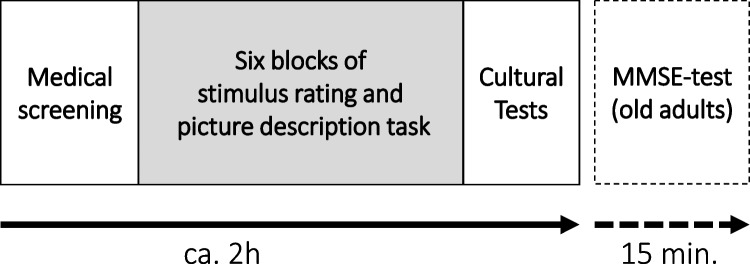
Overview over the experimental procedure

The participants were tested in one session with one to four participants.^4^ Each participant sat in front of a 15.6” laptop at their individual desk. The desks were separated by visual shields to prevent interactions with other participants and the experimenter ensured compliance of the participants. Figure 2 provides an overview about the procedure in each session.

The experiment was written in E-Prime 2.0. An English version of the on-screen and written instructions for the rating task can be found in S4 of the Supplementary Material. Please note that English version of the instructions was translated into German and Chinese in order to provide the localized version of the instructions. The translated versions were checked with each other and the English version to ascertain that the meaning was the same.

**Fig. 3:**
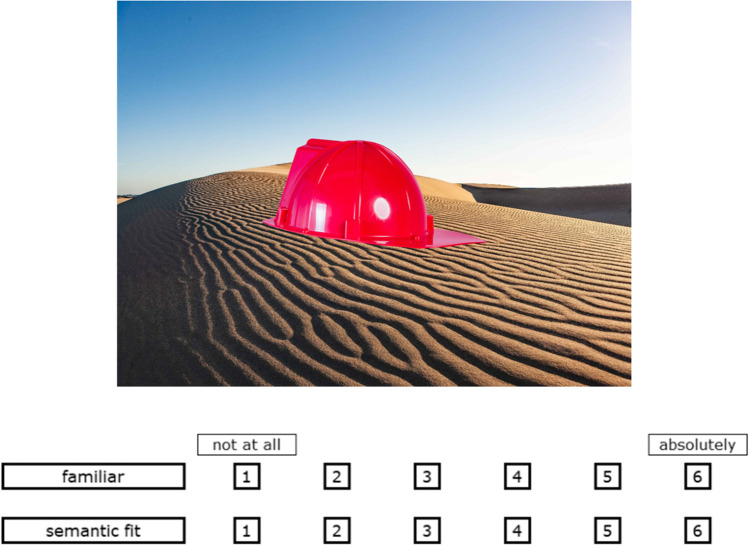
Example of the rating screen

The rating consisted of six blocks with 120 scene–object arrangements per block. The order of the blocks was counterbalanced across participants. Moreover, pictures from the same quadruple were never in the same block to reduce carry-over effects. Each rating trial had the following structure. Each trial started with the presentation of a blank screen for 250 ms. Then, a screen with a scene–object arrangement and two rating scales appeared (Fig. 3). Participants had to rate the object on familiarity and the scene–object arrangement on semantic fit on a six-point scale (1: not at all, 6: absolutely). If an object was unknown to or unrecognizable for the participant, they were to give a familiarity rating of 1. There was no time limit for the rating. After the participants rated the stimulus on both rating scales, a button with the word “next” on it appeared at the bottom of the rating screen. The next trial started once participants clicked on the button. At the end of each block, there was a short, self-determined break and a picture description task. The procedure and results of the description task will be reported elsewhere.

At the end of the rating study, participants completed a German or Chinese translation of the updated version of the SCS (Kitayama et al., 2014; Singelis & Sharkey, 1995).^5^ Older adults additionally completed the MMSE.

### Data analysis

With the first analyses, we provide an overview over the complete picture set using the aggregated values reported in the accompanying Excel table (see also S1 for details). Since researchers typically use such aggregated values from published ratings rather than the participants’ raw values during stimulus selection and as covariates in data analysis (see Weigl et al., 2020 for an example), we treated the quadruples, not the participants, as cases, and the aggregated ratings as the dependent variables. Thus, culture and age group are repeated-measure factors after aggregation (i.e., four aggregated means for each quadruple), even though they were between-subjects factors before aggregation. Such a repeated-measure approach allowed us to provide insights on the characteristics of the whole picture set and to investigate the correlation between the groups in their ratings.^6^

Rating data for each picture were aggregated separately for each age and culture group. In order to assess whether there were some inherent cultural or age differences in the rating scores, a 2 x 2 repeated-measure (rm) ANOVA with the factors Culture (German vs. Chinese) and Age Group (younger vs. older adults) was calculated separately for averaged familiarity and semantic fit of each quadruple. Significant interactions were followed-up with *t* tests for dependent samples. As a manipulation check, one-sample *t* tests for each of the four groups were used to check, if familiarity ratings were above 4 (indicating high familiarity) and semantic fit ratings were lower than 3 (indicating low semantic fit).

Additional aligned rank transform (ART) ANOVAs, a non-parametric, rank-based alternative to factorial rm-ANOVA (Wobbrock et al., 2011), which allows testing not only for main effects, but also for interaction effects within the same analysis by subjecting aligned rank-transformed data^7^ to an ANOVA, and Wilcoxon signed-rank tests were calculated to ascertain the robustness of the parametric analyses. Consistency in the ratings across the groups were investigated with Spearman’s rank correlation.

In addition, and complementary to the repeated-measure approach, we also analyzed the familiarity and semantic fit ratings by means of multilevel linear modeling (MLM; see Tabachnick & Fidell, 2007, for a general introduction). MLM allowed us to consider the hierarchical and nested structure of our data. We used lme4 (D.M. Bates et al., 2015) and lmerTest (Kuznetsova et al., 2017) with the BOBYQA (boundary optimization by quadratic approximation) optimizer for all models in order to increase the likelihood of convergence.

We also analyzed each quadruple individually, i.e., on the level of quadruples. Please note that – unless mentioned otherwise – these analyses were conducted on the raw (i.e., non-aggregated) data for each quadruple. In order to gauge the reliability of the ratings, the familiarity ratings from the first scene were correlated with the ratings from the second scene for each object (e.g., the familiarity ratings of the red helmet in front of dessert 1 were correlated with the familiarity ratings of the very same helmet in front of dessert 2).

For each quadruple, the ratings averaged across the four variants were subjected to a 2 (Culture: Germany vs. China) x 2 (Age Group: Younger vs. Older) between-subject ANOVA separately for familiarity and semantic fit. Thereby, we wanted to find out, which quadruples differ as a function of culture, age, or both.

In addition, we used mixed models^8^ with the between-subjects factors Culture (Germany vs. China) and Age Group (Younger vs. Older adults) and the within-subjects factors Object (Object 1 vs. Object 2) and Background (Background 1 vs. Background 2) as independent variables. The ratings (either familiarity or semantic fit) were the dependent variables. These analyses allowed us to assess for which quadruples object and background versions played a role in addition to culture and age group.

Finally, we wanted to assess whether we were successful in creating object–scene compositions with low and high semantic fit. For this purpose, we analyzed how many of the quadruples with low semantic fit would be classified as low in semantic fit and how many of the quadruples with high semantic fit would be classified as high in semantic fit by all four groups at different criteria (using the mean over participants and versions for each group). A more conservative criterion (<3 for low semantic fit and >4 for high semantic fit) and a more liberal criterion (<3.5 for low semantic fit and >3.5 for high semantic fit) was used for this purpose.

All analyses were conducted in R 4.2.0 (R Core Team, 2022) and RStudio 2022.02.2+485 (RStudio Inc.) using the following central packages WebPower 0.7 (Z. Zhang & Mai, 2022), car 3.1-0 (Fox & Weisberg, 2019), ez 4.4-0 (Lawrence, 2016), tidyverse 1.3.2 (Wickham et al., 2019), Hmisc 4.7-0 (Harrell Jr., 2022), lsr 0.5.2 (Navarro, 2015), effect size 0.7.0 (Ben-Shachar et al., 2020), lme4 1.1-30 (D. M. Bates et al., 2015), lmerTest 3.1-3 (Kuznetsova et al., 2017), nlme 3.1-157 (Pinheiro & Bates, 2000), and ARTool 0.11.1 (Kay et al., 2021)^9^. The code, the completely anonymized data, and the aggregated norms are available on Open Science Framework (OSF: https://osf.io/qx6pf/).

## Results

### Analyses of the complete picture set

**Fig. 4:**
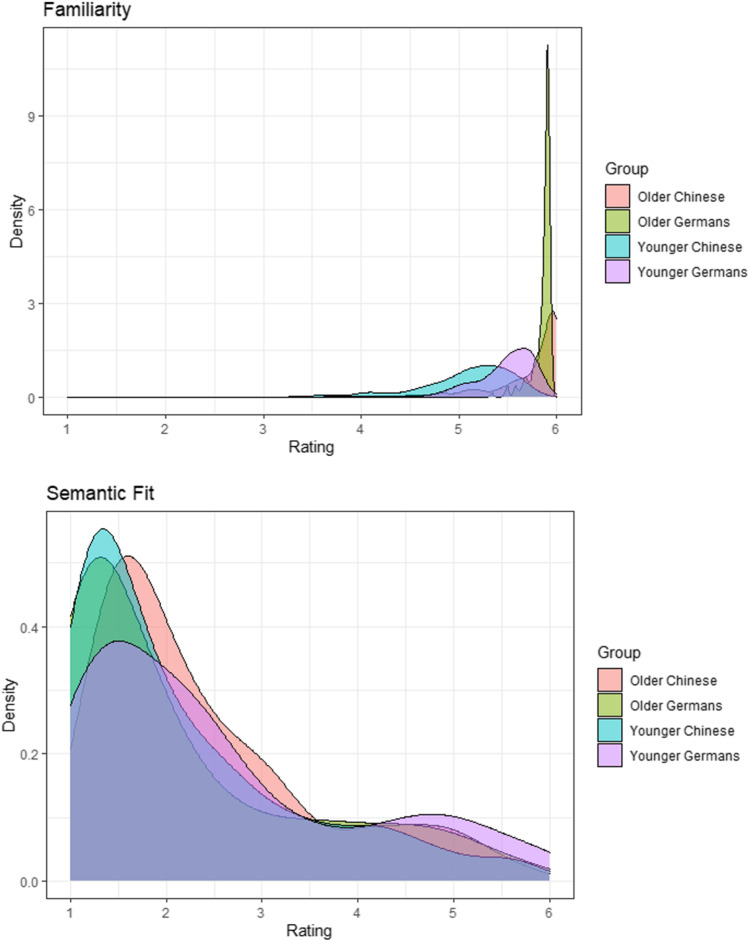
Density plot for the averaged ratings for each quadruple and each rating dimension

In this section, we provide some general characterization of our stimulus material. Summary statistics for the complete picture set can be found in Table 2. Figure 4 depicts the distribution for the averaged ratings of each quadruple. The averaged values can be found in the accompanying Excel table (see S1 for more information on the Excel table).

**Table 2:** Mean (*SD*) familiarity and semantic fit for the complete picture set

	German		Chinese	
	Younger adults	Older adults	Younger adults	Older adults
Familiarity	5.46 (0.35)	5.86 (0.10)	5.09 (0.47)	5.76 (0.34)
Semantic fit	2.53 (1.41)	2.19 (1.29)	2.16 (1.17)	2.41 (1.14)

As intended, the objects were highly familiar to the participants and the majority of quadruples had low semantic fit. Consistent with this visual impression, the one-sample *t* tests revealed that familiarity ratings were above 4 and semantic fit ratings were below 3 (all |*t*s| > 4.5, *p*s < .001) for all four groups. However, the distributions of the ratings were not the same for the four groups (Fig. 4). This visual impression was corroborated by the rm-ANOVAs.

The rm-ANOVA for the familiarity ratings revealed a main effect for Culture (*F*(1, 179) = 96.46, *p* < .001, η_G_^2^ = .11), suggesting higher familiarity ratings in German participants relative to Chinese participants, a main effect for Age Group (*F*(1, 179) = 647.34, *p* < .001, η_G_^2^ = .38), suggesting higher familiarity ratings in older adults relative to younger adults. Moreover, there was an interaction between Culture and Age Group (*F*(1,179) = 154.53, *p* < .001, η_G_^2^ = .04), suggesting that the cultural differences were more pronounced in younger adults relative to older adults. The rm-ANOVA for the semantic fit ratings revealed a main effect for Culture (*F*(1, 179) = 4.19, *p* = .042, η_G_^2^ = .00), but no main effect for Age Group (*F*(1, 179) = 2.52, *p* = .114, η_G_^2^ = .00), and an interaction between Culture and Age Group (*F*(1,179) = 217.70, *p* < .001, η_G_^2^ = .01), suggesting that older Germans gave lower semantic fit ratings than younger Germans, whereas the reverse was true for Chinese participants. The follow-up *t* tests were significant for all comparisons (Table 3), indicating that there are cultural differences within each age group and age-related differences within each culture in the familiarity and semantic fit ratings.

**Table 3:** Results for the follow-up *t* test for familiarity and semantic fit

	Younger vs. older Germans	Younger vs. older Chinese	Younger Germans vs. younger Chinese	Older Germans vs. older Chinese
Familiarity	*t*(179) = –16.90, *p* < .001	*t*(179) = –28.39, *p* < .001	*t*(179) = 12.68, *p* < .001	*t*(179) = 4.38, *p* < .001
Semantic fit	*t*(179) = 10.27, *p* < .001	*t*(179) = –6.46, *p* < .001	*t*(179) = 8.96, *p* < .001	*t*(179) = –5.18, *p* < .001

This pattern was largely corroborated by the non-parametric tests (see S2 in the Supplementary Material). The ART ANOVA replicated all effects except the main effect for Culture in the semantic fit ratings (*p* = .200). All Wilcoxon signed-rank tests were significant (*p* < .001), except for the comparison of the familiarity ratings for German and Chinese older adults (*p* = .188).

Moreover, the results from the MLM essentially replicated the results from the (ART) ANOVAs (see S3 in the supplementary material for details on the model section process and interpretation). In addition, the MLM results indicated that years of education and SCS did not have an impact on the ratings.

Table 4 contains the correlation matrix with the Spearman’s rank correlations for all four groups and both rating dimensions. The ratings are significantly correlated between the four groups for both familiarity and semantic fit. This suggests that the stimuli were perceived similarly across all four groups despite differences in the absolute values. The correlations for familiarity were lower than the correlations for semantic fit. One reason for this difference might be that we allowed variance for semantic fit, but deliberately restricted our material to familiar objects.

**Table 4:** Spearman correlation between the different scales

		Familiarity				Semantic fit			
		Younger Germans	Older Germans	Younger Chinese	Older Chinese	Younger Germans	Older Germans	Younger Chinese	Older Chinese
Familiarity	Younger Germans	-							
	Older Germans	.38*	-						
	Younger Chinese	.66*	.45*	-					
	Older Chinese	.31*	.49*	.61*	-				
Semantic fit	Younger Germans	.17*	.09	.17*	.21*	-			
	Older Germans	.12	.09	.14	.20*	.92*	-		
	Younger Chinese	.15*	.12	.20*	.26*	.91*	.91*	-	
	Older Chinese	.07	.10	.13	.27*	.79*	.86*	.86*	-

*p < .050

Taken together, these results indicate that both cultures and both age groups perceived the stimuli as intended. The objects were highly familiar to the younger and older German and Chinese participants in our sample. Likewise, semantic fit ratings were low, as intended. Furthermore, the ratings were correlated. Despite this overall agreement in the ratings, small, but significant differences between cultures and age groups were observed.

### Analysis for the quadruples in the picture set

Next, we looked at the ratings within each quadruple to assess the comparability of the stimulus material across cultures and age groups. In a first step, we gauged the reliability of the ratings by correlating the ratings for object familiarity with the Spearman correlation. The Spearman correlations for the object familiarity ratings in the first and second scene ranged from .36 to 1.00 (*M* = .78, *SD* = .32)^10^ and were significant for all 360 objects. This points to the reliability of the ratings. Due to many data points with zero variance (especially in the older Chinese) indicating very high consistency in the ratings, group-specific correlations could not be computed. Therefore, we will refrain from reporting group-specific correlations.

The ANOVAs for familiarity and semantic fit on the level of the quadruples were conducted to identify the quadruples, which do not significantly differ as a function of culture, age, or both. The number of significant main effects and interactions can be found in Table 5. As could be expected based on the observed age differences in the aggregated data (Table 2), most (i.e., 158 out of 180) quadruples were associated with significant age differences in the familiarity ratings. The number of significant results for the other main effects and interactions ranged from 20 to 68. Only 13 quadruples did not have any significant effects for familiarity. By contrast, 55 quadruples did not have any significant effects for semantic fit.

**Table 5:** Number of quadruples with significant effects for culture, age, or culture x age in the quadruple-specific 2 x 2 ANOVA

	Culture	Age	Culture x Age
Familiarity	41	158	20
Semantic fit	59	44	68

The number of significant main effects and interactions of the mixed models for familiarity and semantic fit on the level of the quadruples can be found in Table 6. When the object and background versions were also considered, the number of non-significant quadruples dropped to 4 for the familiarity ratings and 15 for the semantic fit ratings. Moreover, there were numerous instances, in which the ratings significantly differed between the object and/or background version (either alone or in interaction with the other factors). However, the average number of main effects and interactions per quadruple were still low (familiarity: *M* = 2.80, *SD* = 1.55, semantic fit: *M* = 2.83, *SD* = 1.78).

**Table 6:** Number of quadruples with significant main effects or interactions in the mixed models

	Familiarity	Semantic fit
Culture	40	60
Age	159	46
Object	84	70
Background	21	63
Culture x Age	20	68
Culture x Object	42	31
Culture x Background	12	23
Age x Object	63	32
Age x Background	11	30
Object x Background	8	22
Culture x Age x Object	22	16
Culture x Age x Background	7	18
Culture x Object x Background	3	10
Age x Object x Background	7	8
Culture x Age x Object x Background	5	13

Moreover, 19 out of 30 quadruples with semantic fit were rated higher than 4 on the semantic fit scale by all four groups and 118 out of 150 quadruples with no semantic fit were rated with less than 3 on the semantic fit scale by all four groups. When using 3.5 as cut-off for the pictures, 23 out of 30 quadruples with semantic fit were rated as fitting and 137 out of 150 quadruples without sematic fit were rated as not fitting by all four groups.

To sum up, we found a high consistency in the familiarity ratings across different scenes. In addition, most of the congruent quadruples were rated high in semantic fit and most of the incongruent quadruples received low semantic fit ratings. These results point to the reliability of the ratings. However, we found significant age differences in the familiarity ratings for the majority of the quadruples and there are only few quadruples without any significant effects.

## Discussion

### The ORCA picture database from a cross-cultural and aging perspective

Standardized, rated stimulus material is important for reproducible research, which can be compared with and transferred to other labs (Souza et al., 2020; Wilcox & Claus, 2017). With the needs of the cross-cultural, aging, and neuroscience communities for rated material in mind, we created a new picture database, which should meet the following five criteria: (1) object–scene compositions with visually and semantically matched distractor for every object and scene, (2) high and low semantic fit between the objects and scenes (3) ratings from younger and older German and Chinese adults, (4) objects familiar to both cultures and age groups, and (5) a large number of stimuli.

Here we presented the ORCA picture database, an extensive collection of 720 object–scene compositions, which were arranged into 180 quadruples, in which each object and background is paired with a semantically and visually matched variant (fulfilling criteria 1 and 5). All compositions were rated for object familiarity and semantic fit between object and scene by younger and older German and Chinese adults (fulfilling criterion 3).

As intended, the objects we presented were highly familiar for all four groups (fulfilling criterion 4). Nevertheless, culture and age affected the familiarity ratings. Similar to Yoon et al. (2004), we found that object familiarity was higher for older adults than for younger adults indicating that older adults have had more lifetime exposure to such objects than younger adults. We also found that object familiarity was higher for German participants than for Chinese participants. This suggests that on average the objects are more typical for Westerners than East Asians despite the careful selection of the objects. In addition, the cultural differences in familiarity were larger for younger than older adults. Given the lower numerical (though not statistically significant) average age of the younger Chinese participants relative to the younger German participants, this suggests that young Chinese participants might have had the least exposure to the objects in our picture data set than the other three groups. This aspect needs to be considered when using the ORCA pictures in cross cultural studies with only younger adults.

The results for the semantic fit indicate that congruent object–background combinations were associated with high semantic fit ratings and incongruent object–background combinations were associated with low semantic fit ratings, as intended (fulfilling criterion 2). However, we again found significant differences between the four groups. Semantic fit was rated lower by German older adults and Chinese younger adults as compared to German younger adults and Chinese older adults. The semantic fit results for younger adults (Chinese < Germans) suggest cultural differences in the perception of incongruency. These results are in line with results from an event-related potential (ERP) study by Goto et al. (2010), who used congruent and incongruent object–background pairings to study sensitivity to incongruency in European and Asian American younger adults. Goto and colleagues focused on the N400, an ERP component sensitive to semantic congruency. They reported that young Asian Americans were more sensitive to incongruent object–background parings than European Americans as evidenced by higher N400 amplitudes. Goto et al. (2010) argued that this cultural difference reflects the higher context-sensitivity of East Asians, because they process their environment to a greater degree than Westerners. Moreover, the higher sensitivity to incongruity was replicated in a subsequent ERP study using face–background pairings (Goto et al., 2013). Together, these studies suggest that the semantic fit results might reflect a higher sensitivity to incongruency in young Chinese relative to young Germans.

Interestingly, we found that the pattern is reversed in older adults (Germans < Chinese). Some evidence suggests that Chinese older adults think more holistically than younger Chinese adults or American adults irrespective of age (X. Zhang et al., 2014). In fact, and in line with this reasoning, we found that Chinese older adults had a more interdependent self-construal than the remaining three groups. This might suggest that the more holistic thinking of Chinese older adults enabled them to reconcile the incongruency presented in the object–background combination. However, these interpretations of the semantic fit results, both for younger and older adults, are speculative at present. More systematic, confirmatory research is required to critically test the validity of these interpretations.

The MLM suggests that differences in years of education or self-construal did not play a major role in the ratings. At first sight, it seems surprising that SCS, for which cultural differences are reported in the literature (e.g., Singelis & Sharkey, 1995; Yoon et al., 2000) and were found in the present sample, did not account for variance in the ratings, even though culture had an influence on the prediction. However, the SCS consists of questions about the self and its relation to other people. The stimulus material, by contrast, consist of objects or animals placed on a background scene and the rating scales do not involve the self. Thus, it might be the case that the type of cultural differences assessed by the SCS were not relevant for the familiarity and semantic fit ratings. Other questionnaires such as the Analytic-Holistic-Scale (Choi et al., 2007), which more broadly assess differences between analytic and holistic cognition, might have been more suitable for the rating data at hand.

The absence of an influence of years of education on the ratings might indicate that all participants were sufficiently educated for the rating task at hand. This might well be the result from our recruitment strategy, i.e., we recruited from an educated participant pool, which is also most likely to participate in psychological studies in general. However, the results for years of education must be treated with caution, because some German participants misinterpreted the question on years of education. Future studies might provide more detailed explanation on this question in order to achieve cross-culturally comparable data on education. However, the absence of statistically significant influences of years of education and SCS is reassuring, because it suggests that our reported ratings are in fact only affected by the variables of interest, namely age and culture.

Many aging studies test age differences in associative memory for pictorial stimuli without reporting that the material to be associated has been rated for familiarity and/or semantic fit by all age groups under investigation (e.g., Guez & Lev, 2016; Naveh-Benjamin et al., 2003). A similar case can be made for cross-cultural studies (e.g., Masuda & Nisbett, 2001). The results from the ORCA rating suggest that it might be dangerous to simply rely on the researchers’ judgments or to collect only ratings for one group (e.g., younger adults) and assume equivalence across the remaining groups under investigation. Moreover, the same object was perceived differently depending on the surrounding background (Davenport, 2007; Palmer, 1975). This highlights the need of rating objects and background in combination rather than in isolation.

Although the cultural and age differences in familiarity and semantic might be problematic for some researchers, who want to control for these factors by achieving non-significance in the selected stimuli. In actual cross-cultural studies with different age groups, however, these differences might be less problematic, because group-specific ratings for the stimuli can be included in the statistical modeling process (e.g., with MLM; Tabachnick & Fidell, 2007).

To sum up, we found that the general direction of the ratings was similar across cultures and age groups and in the intended direction (i.e., high overall object familiarity and low semantic fit for the majority of the stimuli). However, small yet significant culture and age differences emerged. Of note, there was a high consistency in the findings across the different analysis schemes (parametric tests, non-parametric tests, and MLM) pointing to the robustness and reliability of our results.

### Benefits of the ORCA picture database

There are several benefits associated with the ORCA picture set. First of all, every participant provided ratings for object familiarity and semantic fit. As revealed by the correlation analyses, these ratings have a relative high consistency on average. The extensive ratings allow researchers to select the items with the level of consistency needed for their research.

Second, we provide a large number of stimuli. This is especially advantageous for neuroscientific research, which often requires large number of trials to obtain a satisfactory signal-to-noise ratio (e.g., Luck, 2014). Another advantage especially for neuroscientific research is that all objects are centered in the middle of the scene, which helps reducing eye-movement and consequently artifacts. This makes our picture set especially relevant for the emerging field of cultural neuroscience (Denkhaus & Bös, 2012), which looks for cultural differences in brain activity in general (Han & Ma, 2014) or in conjunction with aging (Gutchess & Huff, 2016). Of course, the ORCA picture set can also be used in cognitive research on culture and/or aging without a neuroscience aspect.

Third, our picture database adds to the growing number of stimulus sets containing ratings for culture and age. As stimulus sets normed for a particular group are often transferred to other samples without much regard for potential cultural or age differences (cf. Yoon et al., 2004), material rated for several subpopulations helps to increase the reliability and reproducibility of psychological research.

Fourth, each quadruple contains objects and backgrounds with high visual and semantic similarity. This makes ORCA perfectly suited for memory research, which requires matched targets and lures to prevent that strategic retrieval strategies bias memory measures. Moreover, high target-lure similarity requires to build up detailed memory representations and hippocampus-based (pattern separation) processing, a hallmark of episodic memory (Stark et al., 2019). Of course, ORCA is also suitable for any research which profits from strongly matched stimuli for counterbalancing (e.g., studies on visual scene processing).

Last, but not least, we provide not only aggregated data on the ORCA database, but also make the raw data and R code freely available on OSF. This gives other researchers the maximal amount of information to decide for themselves, whether the ORCA stimulus material fits their research purposes. Moreover, we provide the exact instructions for our rating study, which enables other researchers to extend on our work and collect ratings for other populations.

### Limitations

The ORCA picture database also has a few limitations. First of all, the background has not been rated for familiarity. However, as the objects were central for our intended memory study, object familiarity was particularly important to us. Object familiarity was collected twice, allowing us to assess whether and how the background scene changed the feeling of familiarity for the depicted objects. The results from the correlation analyses between the familiarity ratings on different scenes suggest a high reliability of the familiarity ratings. Ratings on the familiarity of the background scene could be collected in future studies.

Second, there are no ratings for the spatial fit of the objects in the background scene. Such ratings would be useful, because the objects were placed at the center of the picture with little regard to spatial fit. However, we will collect ratings of spatial fit for the majority of pictures in a future project and append this information to the ORCA database.

A third limitation is the lack of ratings for pleasantness and arousal, which are often collected in rating studies (Souza et al., 2020). While there might be some variability in pleasantness and arousal, we paid attention to choosing neutral and low-arousing objects and scenes. Thus, our picture set is suitable for research questions requiring neutral stimuli (e.g., studies on source memory). However, ratings for pleasantness and arousal could be collected in future studies.

Fourth, we sampled our German and Chinese participants from the university student population for younger adults and an educated general population for older adults. This sampling strategy was chosen, because these populations typically participate in psychological experiments. Thus, the samples we tested are not representative for the general populations. It is known in the literature that most participants in psychological studies are not representative for the general population (e.g., participants are more educated relative to the population; Henrich et al., 2010). For the very same reason, most rating studies rely on student populations. As a case in point, more than 76% of the rating studies reviewed by Souza et al. (2020) recruited only university students. Thus, the ORCA ratings are most useful for researchers who rely the participant population most common in cross-cultural and/or aging research and who are interested in comparing Germans and Chinese. Our ratings are less applicable for the general (non-academic) population, more specific subpopulation (e.g., clinical populations), or non-German/non-Chinese cultures. Future studies might use online ratings to extend upon our ratings to different populations. In order to facilitate such studies, we provide the complete instructions in the supplement.

## Conclusions

With the ORCA picture database, we provide a stimulus set normed for object familiarity and semantic fit in younger and older German and Chinese participants. The large number of visually and semantically matched object–scene combinations makes this picture set ideally suited for neuroscientific research on culture, age and the interaction between both.

## Supplementary information

ESM 1 (DOCX 1.42 MB)

## Footnotes

1 Sample size was determined via power analysis using the R package WebPower (Z. Zhang & Mai, 2022). Since large effects in the quadruple specific analyses of variance (ANOVAs) were of most interest to us (see section 2.4 for details), we conducted the power analysis with f = .40, α = .05, and 1-β = .95. The recommended overall sample size was 83. In consideration of practical aspects such as counterbalancing of the picture lists and the total duration of the experimental session (see section 2.3 for details), we settled on a sample size of 96 (24 participants per group). No participant was dropped from analysis, but in two cases, testing had to be aborted due to non-eligibility (*n* = 1 due to Morbus Best, *n* = 1 due to having both, a Western and an East Asian cultural background). Please note that institutional regulations after the onset of the COVID-19 pandemic prevented us from completing the sample of the older German adults. Therefore, there are only 23 older German adults.

2 Please note that this database is no longer accessible via the link, but can be obtained by contacting Joshua Goh directly.

3 The terms of use can be found here: https://www.uni-saarland.de/fileadmin/upload/lehrstuhl/mecklinger/Dokumente/readme_-_ORCA.txt.

4 The data from German adults were collected between December 2019 and March 2020. The data from Chinese adults were collected between December 2019 and August 2020. Due to the onset of the COVID-19 pandemic in early 2020, most participants were tested alone. Younger German adults were tested in sessions with 1–4 participants and older German adults were tested in session with 1–2 participants. All Chinese participants were tested alone.

5 In addition to the SCS, we also included the pen test (Kim & Markus, 1999), which measures preference for harmony vs. individuality, and an abridged version of the Framed Line Test (Kitayama et al., 2003), which non-verbally measures attention to the context to explore their sensitivity for assessing cultural differences in future studies. Since these exploratory tests are not relevant for the current study and were not sensitive to cultural differences (all *p*s > .307), they will not be further discussed.

6 Scariano and Davenport (1987) showed that type I error rate becomes drastically inflated if correlated data are subjected to a between-subject ANOVA. This additionally underscores the need for a repeated-measure approach.

7 The aligned rank transform corrects for problems in factorial analyses with conventional rank transforms by first aligning the data for each main effect and interaction and then assigning ranks (cf. Wobbrock et al., 2011).

8 Although technically the mixed models are also multilevel models, we distinguish between both terminologically. The mixed models were used as an alternative to mixed-design ANOVAs, because mixed models produced results equivalent to mixed-design ANOVAs (Field et al., 2012) and were more efficient to use in R. Moreover, we only interested in the analysis of variance table containing the F statistic for main effects and interactions.

9 Complete information on the R sessions can be found in the session information at the end of the HTML documents on OSF.

10 As the Spearman correlation is actually a Pearson correlation for ranks, mean and standard deviation were computed after using Fisher’s *z*-transform. The results were back-transformed to *r* coefficients for interpretation.
