# Supplementary material for: ORCA: A picture database of object–scene arrangements for cross-cultural and aging research
Source: Behav Res Methods. 2023 Jan 26;56(2):513–28. doi: 10.3758/s13428-023-02064-x (PMC9879563; doi:10.3758/s13428-023-02064-x)
Supplement: Supplementary file 1 — (DOCX 1.42 MB) [file 13428_2023_2064_MOESM1_ESM.docx]

**Supplementary material**

**S1. Organization of the Excel file with the aggregated data.**

The Excel file with the picture norms can be found on OSF:

<https://osf.io/qx6pf/>

Below we list all sheets and all variables of the accompanying Excel file and their interpretation

**S1.1 Sheet “ORCA_Ratings”**

**Region:** Region of the sample (Germany or China)

**AgeGroup:** Age group of the sample (younger vs. older)

**Picture:** Number of the quadruple

**Semantic_Congruency:** Whether the quadruple was semantically congruent or incongruent

**N_Famil, N_Seman:** Number of participants in the sample

**meanFamil_1a, meanFamil_1b, meanFamil_2a, meanFamil_2b, sdFamil_1a, sdFamil_1b, sdFamil_2a, sdFamil_2b, medianFamil_1a, medianFamil_1b, medianFamil_2a, medianFamil_2b,** **lowerCI_Famil_1a, lowerCI_Famil_1b, lowerCI_Famil_2a, lowerCI_Famil_2b, upperCI_Famil_1a, upperCI_Famil_1b, upperCI_Famil_2a, upperCI_Famil_2b:** Mean, standard deviation, median, and lower and upper bound of the 95% confidence interval of the mean for the familiarity ratings of each picture version of a quadruple

**meanSeman_1a, meanSeman_1b, meanSeman_2a, meanSeman_2b, sdSeman_1a, sdSeman_1b, sdSeman_2a, sdSeman_2b, medianSeman_1a, medianSeman_1b, medianSeman_2a, medianSeman_2b, lowerCI_Seman_1a, lowerCI_Seman_1b, lowerCI_Seman_2a, lowerCI_Seman_2b, upperCI_Seman_1a, upperCI_Seman_1b, upperCI_Seman_2a, upperCI_Seman_2b:** Mean, standard deviation, median, and lower and upper bound of the 95% confidence interval of the mean for the semantic fit ratings of each picture version of a quadruple

**N_Unfamiliar_1a, N_Unfamiliar_1b, N_Unfamiliar_2a, N_Unfamiliar_2b:** Number of participants, for whom the pictures were not familiar at all (i.e., gave a rating of 1)

**quadrupleFamil, quadrupleSeman:** Average of the ratings for each of the pictures in the quadruple

**rangeFamil, rangeSeman:** Range for the ratings for each picture in the quadruple for familiarity and semantic fit

**pFamil.Culture, pFamil.Age, pFamil.Interaction:** p-values for the main effects for culture and age as well as the interaction from the ANOVA on the average ratings for the quadruple for the familiarity ratings

**pSeman.Culture, pSeman.Age, pSeman.Interaction:** p-values for the main effects for culture and age as well as the interaction from the ANOVA on the average ratings for the quadruple for the semantic fit ratings

**S1.2 Sheet “ORCA_Mixed_Model_Results”**

**Picture:** Number of the quadruple

**pFamil.Culture, pFamil.Age, pFamil.Object, pFamil.Scene, pFamil.Culture_Age, pFamil.Culture_Object, pFamil.Age_Object, pFamil.Culture_Scene, pFamil.Age_Scene, pFamil.Object_Scene, pFamil.Culture_Age_Object, pFamil.Culture_Age_Scene, pFamil.Culture_Object_Scene, pFamil.Age_Object_Scene, pFamil.Culture_Age_Object_Scene:** p-values for the main effects for culture, age, object (front object variant), and scene (background variant) as well as all possible interactions between these factors from the mixed model analysis on the raw ratings for the quadruple for the familiarity ratings

**pSeman.Culture, pSeman.Age, pSeman.Object, pSeman.Scene, pSeman.Culture_Age, pSeman.Culture_Object, pSeman.Age_Object, pSeman.Culture_Scene, pSeman.Age_Scene, pSeman.Object_Scene, pSeman.Culture_Age_Object, pSeman.Culture_Age_Scene, pSeman.Culture_Object_Scene, pSeman.Age_Object_Scene, pSeman.Culture_Age_Object_Scene:** p-values for the main effects for culture, age, object (front object variant), and scene (background variant) as well as the interactions between these factors from the mixed model analysis on the raw ratings for the quadruple for the semantic fit ratings

**S1.3 Sheet “ORCA_Physical_Properties”**

**Quadruple number:** Number of the quadruple

**Version:** Specific object-scene combination of the quadruple

**luminance mean:** Average luminance of the specific picture

**contrast (luminance std dev):** Contrast based on luminance distribution

**CIELAB L*, CIELAB a*, CIELAB b*:** Values for perceptual lightness (L*), green-red (a*), and blue-yellow (b*) coordinates in the CIELAB color space

NOTE: Luminance mean, contrast, and CIELAB coordinates were computed with GIMP 2.10.18 (www.gimp.org).

**S2. Non-parametric tests for the analysis of complete picture set**

The ART ANOVA for the familiarity ratings revealed a main effect for Culture (*F*(1, 179) = 284.40, *p* < .001), a main effect for Age Group (*F*(1, 179) = 1155.00, *p* < .001), and an interaction between Culture and Age Group (*F*(1,179) = 279.87, *p* < .001). The ART ANOVA for the semantic fit ratings revealed neither a main effect for Culture (*F*(1, 179) = 1.65, *p* = .200) nor a main effect for Age Group (*F*(1, 179) = 0.40, *p* = .527). The interaction between Culture and Age Group was significant (*F*(1,179) =221.45, *p* < .001). The follow-up Wilcoxon signed-rank tests were all significant except for one comparison (Table S1).

Table S1. Results for the Wilcoxon signed-rank tests for familiarity and semantic fit.

|  | Young vs. old Germans | Young vs. old Chinese | Young Germans vs. young Chinese | Old Germans vs. old Chinese |
| --- | --- | --- | --- | --- |
| Familiarity | p < .001 | p < .001 | p <.001 | p = .188 |
| Semantic Fit | p < .001 | p < .001 | p <. 001 | p < .001 |

**S3. Multilevel Linear Modeling of the Rating Data**

**S3.1 Methodological Details on the Multilevel Linear Modelling Approach**

Separate models were estimated for familiarity and semantic fit ratings. In a first step, we defined a baseline model by adding random effects for participants, for quadruples, and for picture variants which were nested within quadruples. Next, fixed effects were added to the model.

For the MLM of the familiarity ratings, we added culture (contrast-coded: -1 Germany, 1 China), age (grand-mean centered on participant level), the interaction between culture and age, and semantic fit ratings (group-mean centered; i.e. centered around each participant’s mean) as fixed effects in the model. For the MLM of the semantic fit ratings, we added culture (contrast-coded: -1 Germany, 1 China), age (grand-mean centered on participant level), the interaction between culture and age, congruency (i.e. whether quadruple was semantically congruent or not; contrast-coded: -1 incongruent, 1 congruent), and familiarity ratings (group-mean centered; i.e. centered around each participant’s mean) as fixed effects in the model.

These models were compared with their respective baseline model and random-slopes were added if the addition would improve model fit as indicated by model comparisons. The best-fitting model was selected for further inspection. Finally, we explored the influence of independent self-construal (SCS), interdependent SCS, and years of education (all grand-mean centered on the participant level). Due to a missing value for years of education, we added independent and interdependent SCS to the best-fitting models and analyzed influence of independent and interdependent SCS, and years of education in separate models based on a sample without missing values. The model selection process with be described in more detail in the next two sections.

**S3.2 Multilevel Linear Model for Familiarity Ratings**

Here, we describe the model selection process for the familiarity ratings. Fit indices for the different models can be found in Table S2 (Top). First, the baseline model was estimated with the familiarity ratings as the outcome variables and random intercepts for participants, quadruples, and pictures variants. The intraclass correlation (ICC) was .438 for all random effects, suggesting that 44% of the total variance was accounted by participants, quadruples, and pictures variants.

Adding culture, age, the interaction between culture and age, and semantic fit ratings as fixed effects significantly improved model fit relative to the baseline model (χ²(4) = 630.88, *p* < .001). Moreover, adding random-slopes (i.e. allowing slopes for the relationship between familiarity and semantic fit to vary across participants) significantly improved model fit relative to the random intercept model (χ²(2) = 1490.50, *p* < .001).

Moreover, including independent and interdependent SCS did not improve the model (χ²(2) = 0.00, *p* = 1.00). The same was true when years of education and the two SCS variables were added to the same random-slopes model based on a reduced data set (i.e. excluding missing values for years of education, but with the same fixed and random effects as above)(χ²(3) = 0.00, *p* = 1.00). This indicates that the model without these covariates is superior in predicting familiarity ratings as compared to the model with these covariates. Therefore, the random-slopes model without covariates was chosen for interpretation.

The fixed effect estimates can be found in Table S3 (Top). Age predicted familiarity ratings, i.e. participants with higher chronological age rated the objects as more familiar. While culture itself did not predict familiarity ratings, culture moderated the relationship between age and familiarity. For Chinese participants, the relationship between age and familiarity was stronger than for German participants. Moreover, familiarity ratings increased with higher semantic fit ratings. In addition, random intercepts and random slopes were negatively correlated (*r* = -.84) indicating that the relationship between semantic fit and familiarity became weaker for participants with higher familiarity ratings.

**S3.3 Multilevel Linear Model for Semantic Fit Ratings**

Here, we describe the model selection process for the semantic fit ratings. Fit indices for the different models can be found in Table S2 (Bottom). We again started with estimating the baseline model. The intraclass correlation (ICC) was .536 for all random effects, suggesting that 54% of the of the total variance was accounted by participants, quadruples, and pictures variants.

Adding culture, age, the interaction between culture and age, congruency, and familiarity ratings as fixed effects significantly improved model fit relative to the baseline model (χ²(5) = 830.55, *p* < .001). Moreover, adding random-slopes (i.e. allowing slopes for the relationship between familiarity and semantic fit to vary across participants) significantly improved model fit relative to the random intercept model (χ²(2) = 176.11, *p* < .001).

Again, including independent and interdependent SCS did not improve the model (χ²(2) = 0.00, *p* = 1.00). The same was true when years of education was additionally considered in a model based on the reduced sample (χ²(3) = 0.00, *p* = 1.00). This indicates that the model without these covariates is superior in predicting semantic fit ratings as compared to the model with these covariates. Therefore, the random-slopes model without covariates was chosen for interpretation.

The fixed effect estimates can be found in Table S3 (Bottom). Semantic fit ratings were higher for congruent object-background pairings than for incongruent object-background pairings. While neither culture nor age themselves predicted semantic fit ratings, culture moderated the relationship between age and semantic fit. For Chinese participants, the relationship between age and semantic fit was positive, i.e. semantic fit ratings were higher for older participants. For German participants, this relationship was negative, i.e. semantic fit ratings were lower for older participants. Moreover, familiarity ratings increased with higher semantic fit ratings. In addition, random intercepts and random slopes were positively correlated (*r* = .42), indicating that the relationship between familiarity and semantic fit became stronger for participants with higher semantic fit ratings.

Table S2. Information on model fit for the multilevel linear model analyses for the familiarity and semantic fit ratings.

|  | AIC | BIC | Log-Likelihood |
| --- | --- | --- | --- |
| Familiarity |  |  |  |
| Baseline Model | 165727 | 165773 | -82859 |
| Model 1 (Random Intercept) | 165104 | 165186 | -82543 |
| Model 1 (Random Slope) | 163618 | 163718 | -81798 |
| Model 2 (with SCS) | 163627 | 163745 | -81800 |
| Model 1* (Random Slope) | 162504 | 162604 | -81241 |
| Model 2* (with Years of Education and SCS) | 162522 | 162649 | -81247 |
| Semantic Fit |  |  |  |
| Baseline Model | 226607 | 226653 | -113299 |
| Model 1 (Random Intercept) | 225787 | 225878 | -112883 |
| Model 1 (Random Slope) | 225615 | 225724 | -112795 |
| Model 2 (with SCS) | 225625 | 225753 | -112798 |
| Model 1* (Random Slope) | 223439 | 223548 | -111707 |
| Model 2*(with Years of Education and SCS) | 223457 | 223594 | -111713 |

* These models are based on a reduced sample (i.e. one participant with missing values for years of education was removed from the sample). Therefore, the relative fit indices of these models also cannot be compared with the relative fit indices of the models based on the complete sample.

Table S3. Information on the fixed effects of the selected models for the familiarity and semantic fit ratings.

|  | *B* | *SE B* | *t* and *p* |
| --- | --- | --- | --- |
| Familiarity |  |  |  |
| (Intercept) | 5.539 | 0.065 | t(101.37) = 85.74, p < .001 |
| Culture | 0.020 | 0.036 | t(89.96) = 0.56, p = .580 |
| Age | 0.005 | 0.001 | t(89.81) = 3.15, p = .002 |
| Semantic Fit | 0.065 | 0.009 | t(97.91) = 7.43, p < .001 |
| Culture x Age | 0.003 | 0.001 | t(89.67) = 2.01, p = .048 |
| Semantic Fit |  |  |  |
| (Intercept) | 3.237 | 0.078 | t(267.29) = 41.34, p < .001 |
| Culture | -0.042 | 0.044 | t(94.89) = -0.95, p = .344 |
| Age | -0.001 | 0.002 | t(94.91) = -0.48, p = .635 |
| Familiarity | 0.137 | 0.013 | t(78.29) = 10.82, p < .001 |
| Congruency | 1.367 | 0.063 | t(178.01) = 21.60, p < .001 |
| Culture x Age | 0.005 | 0.002 | t(94.38) = 2.85, p = .005 |

**S4. Instructions for the rating task**

There were three types of instructions: On-screen instructions, instruction notes, and an FAQ. The on-screen instructions are the instructions in the computerized rating task. Alongside reading the Instruction Rating Screen, all participants read the instruction notes. An FAQ provided standardized responses for questions of the participants.

**S4.1 On-screen Instructions**

**Welcome Screen**

Dear participant!

Thank you very much in advance for participating in our rating study!

In the following screens, we will give you detailed instructions on how to rate the material. Please read these instructions carefully.

If you have any questions, please feel free to ask the experimenter at any time. Continue by pressing SPACE!

**Instruction Rating Screen**

In this rating study, we will present you several pictures.

In every picture, there is an object in front of a scene. We want you to rate these pictures on two attributes.

1) How familiar is the depicted object for you? You can select a number from 1 (not familiar at all) to 6 (absolutely familiar).

2) How well does the object fit into the scene? Again, you can select a number from 1 (does not fit in at all) to 6 (absolutely fits in).

Please use the mouse for making your choices.

We are most interested in your first impression. Therefore, please base your rating on your first impression or gut feeling.

You can proceed to the next picture, once you have selected a number for both attributes.

Do not haste, but also do not spent too much time on a single picture.

There will be breaks on a regular basis.

If you have any questions, please feel free to ask the experimenter.

If you are ready to go, press SPACE!

**Break Screen**

Now you can take a short break.

When you feel ready to continue, press SPACE.

**Thanks screen**

This is the end of the rating study.

Thank you very much for you participation!

Please contact the experimenter!

**S4.2 Instruction Notes**

What is meant by semantic fit?

By semantic fit, we mean that you would associate the depicted objects with the scene depicted in the background or that you would expect to see the object in this context. Important: Fit does not refer to spatial fit.

For example, the objects in these two pictures fit quite well into their surrounding, because we would expect to see a soap in the bathroom and files in an office.


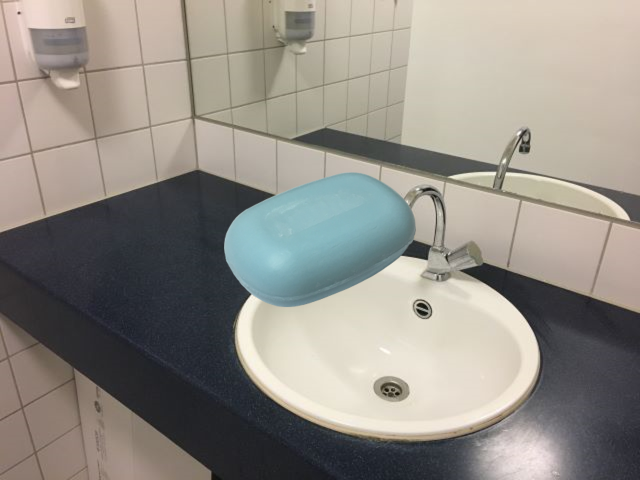

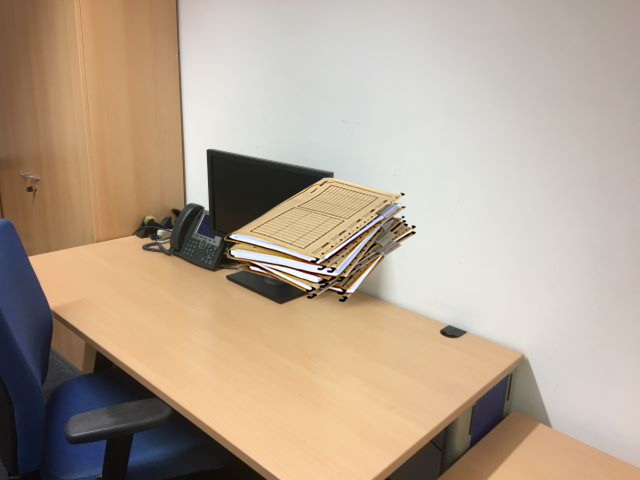


| 1 | 2 | 3 | 4 | 5 | 6 |  |  | 1 | 2 | 3 | 4 | 5 | 6 |
| --- | --- | --- | --- | --- | --- | --- | --- | --- | --- | --- | --- | --- | --- |

In the pictures below, however, the objects fit less well into the surrounding context, because files rarely lie around in a bathroom and soap is rarely found on an office desk.


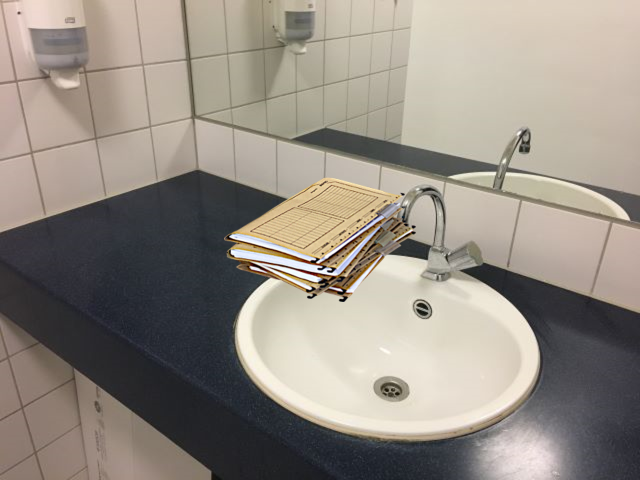

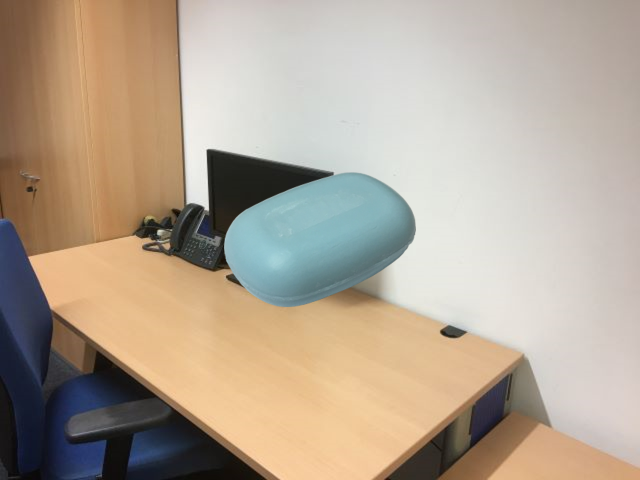


| 1 | 2 | 3 | 4 | 5 | 6 |  |  | 1 | 2 | 3 | 4 | 5 | 6 |
| --- | --- | --- | --- | --- | --- | --- | --- | --- | --- | --- | --- | --- | --- |

What is meant by familiarity?

By familiarity, we mean that you have come across the object (experience in your personal life or exposure via media).

For example, you are most likely familiar with the banana on the left, but not with the object (a fruit peeler) on the right. Therefore, the banana would receive a high number on the rating (indicating very high familiarity), whereas the object on the right would receive a low number (indicating low/no familiarity).


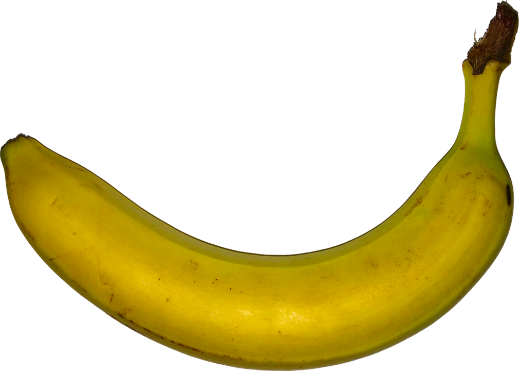

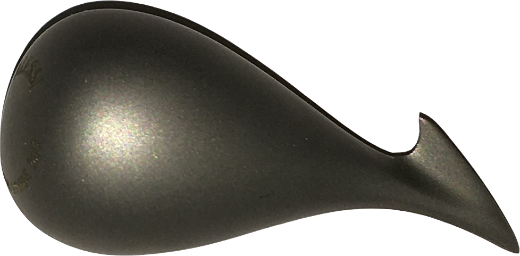


| 1 | 2 | 3 | 4 | 5 | 6 |  |  | 1 | 2 | 3 | 4 | 5 | 6 |
| --- | --- | --- | --- | --- | --- | --- | --- | --- | --- | --- | --- | --- | --- |

What should I answer if an object is completely unknown (or unidentifiable)?

Please answer with “1” on the familiarity rating. For the rating of semantic fit, please answer according to your first impression.

**S4.3 FAQ**

Is there a time limit for the rating?

No, there is no time limit. However, we will present you a lot of pictures and are most interested in your first impression. Therefore, do not spend too much time on a single picture.

How long can I take a break?

You can take a break until you feel fit enough. However, the break should not take much longer than five minutes.
